# Experimental Aspects of Colloidal Interactions in Mixed Systems of Liposome and Inorganic Nanoparticle and Their Applications

**DOI:** 10.3390/ijms130911610

**Published:** 2012-09-17

**Authors:** Raphael Michel*, Michael Gradzielski*

**Affiliations:** Stranski-Laboratorium für Physikalische und Theoretische Chemie, Institut für Chemie, Technische Universität Berlin, Berlin D-10623, Germany; E-Mails: raphael.michel@mailbox.tu-berlin.de (R.M.); michael.gradzielski@tu-berlin.de (M.G.); Tel.: +49-30-314-22822 (R.M.); +49-30-314-24934 (M.G.); Fax: +49-30-314-26602 (M.G.).

**Keywords:** liposome, bilayer, nanoparticle, encapsulation, decorated vesicles, internalization, supported lipid bilayer, small unilamellar vesicle (SUV), colloidal stabilization

## Abstract

In the past few years, growing attention has been devoted to the study of the interactions taking place in mixed systems of phospholipid membranes (for instance in the form of vesicles) and hard nanoparticles (NPs). In this context liposomes (vesicles) may serve as versatile carriers or as a model system for biological membranes. Research on these systems has led to the observation of novel hybrid structures whose morphology strongly depends on the charge, composition and size of the interacting colloidal species as well as on the nature (pH, ionic strength) of their dispersing medium. A central role is played by the phase behaviour of phospholipid bilayers which have a tremendous influence on the liposome properties. Another central aspect is the incorporation of nanoparticles into vesicles, which is intimately linked to the conditions required for transporting a nanoparticle through a membrane. Herein, we review recent progress made on the investigations of the interactions in liposome/nanoparticle systems focusing on the particularly interesting structures that are formed in these hybrid systems as well as their potential applications.

## 1. Introduction

Nowadays, the use of nanoparticles in biotechnology is of major interest due to the amazing potential displayed by mixed systems combining the properties of materials and the specific architectures and functions of the biological world. In particular, nanoparticles are attractive as they exhibit strongly size-dependent properties [1–3], which allows their use as functional system components, as for instance in sensor applications or as delivery vehicles [4–6]. However, they are now also in abundant use as antimicrobial agents (e.g., Ag nanoparticles) [7,8] or in the case of silica nanoparticles for various purposes, such as biomarkers for controlled release [9–11].

In the past few years, intensive research activities have been devoted to the characterization of the interaction between hard nanoparticles and biomolecules among which are proteins [12], DNA molecules [13] and phospholipid molecules (typically in the form of bilayers) [14]. This is an important issue in the context of using nanoparticles in nanomedicine but also in the related field of nanotoxicology [15–18]. Accordingly, fundamental studies in this field are necessary in order to gain a systematic understanding of the relevant aspects in the interactions between nanoparticles and lipid bilayers.

### 1.1. Properties of Phospholipid Bilayers

Phospholipids are amphiphilic molecules composed of a hydrophilic headgroup (phosphate) and two hydrocarbon chains thereby yielding to a packing parameter value *p* [19–21] between 0.5 and 1, granting them a truncated cone form (*p* being defined as: *p = v*/(*al*); with *v* being the volume of the hydrophobic chains, *a* the head group area of the lipid and *l* its stretched length [19]). Thus, phospholipids have a tendency for the formation of bilayers [22], also in the form of closed bilayers, *i.e.*, vesicles [23,24].

Phospholipid vesicles often called liposomes are frequently used in cosmetic and pharmaceutical formulations. This is mostly due to their ability to solubilise hydrophobic compounds and to allow for rheological control of the formulations [25,26]. Liposomes are also known to have a significant potential as drug carriers [27,28]. This is the case as they are able to transport hydrophobic molecules in their bilayer and/or hydrophilic molecules in their interior, thereby being very flexible carrier systems. In addition, having cell membrane mimetic surfaces, they facilitate the transport through membranes while being non cytotoxic and may serve as model systems for experimental and theoretical studies on cell membranes.

Pure phospholipid bilayer membranes exhibit a sharp main phase transition [29] which is lipid specific and corresponds to the melting of their hydrocarbon chains. Below the chain melting temperature (*T*_m_), the phospholipid molecules are locked in place [30], bound tightly together by the van der Waals forces between their hydrocarbon chains, so that the bilayer is in a “solid-state” (gel phase), leading to the appearance of characteristic angular shapes in the case of vesicles [31]. On the contrary, at temperatures above *T*_m_, the lipids exhibit lateral as well as inter-layer mobility (flip-flop [32]) responsible for the fluidity of the membrane. When passing through its phase transition, the bilayer vesicle membrane experiences a temporary permeability allowing the release of molecules from its interior [33]. This last property being one other reason why liposomes are used as nanocontainers in drug delivery technologies, since they enable the achievement of on-demand release of encapsulated molecules by passing through the phase transition temperature.

### 1.2. Methods of Vesicle Formation

The formation of liposomes occurs naturally but in many circumstances is done using appropriate procedures. Phospholipids dispersed in water “spontaneously” form giant multilamellar vesicles (MLVs) which are huge enough to be assimilated to a lamellar phase (it should be noted that typically during dispersion shear is applied, which is mainly responsible for the formation of the vesicles from these lamellar dispersions). However, starting from an MLV suspension, the preparation of well-defined small unilamellar vesicle (SUV) dispersions of specific composition, size and polydispersity can be relatively easily achieved using one or a combination of the different available techniques among which are sonication, rehydration of lipid film, or extrusion [24,25,28,34].

These preparation processes are required since the normal equilibrium state of phospholipids is a lamellar phase, whereas the vesicles are only metastable [35]. Hence, this imposes one limitation to the possible applications of phospholipid vesicles as they are intrinsically unstable, while it might be noted that in some surfactant systems spontaneous vesicle formation and thermodynamic stability are present, especially in the case of cationic surfactants or mixtures of surfactants [36–39].

Thus, the investigation on mixed systems nanoparticles/liposomes is not only of interest from a fundamental point of view, as their interaction leads to novel colloidal structures such as decorated vesicles [40,41] and internalized particles [42] but also due to their innovative potential applications for particle transport, cytotoxicity evaluation and liposome stabilization [40,43].

This review is organized as follows. We first focus on the study of the interactions present in mixed systems liposome/nanoparticle and the various colloidal structures they generate. Particular attention is given to the influence of the phase behaviour of the lipid bilayer (fluid/gel) on the nature of its interaction with colloidal particles. In the second part we present some interesting examples of applications and innovative technologies originating from specific features of these mixed systems. In the last part a particular emphasis is put on the use of silica, gold and silver nanoparticles, as being relevant examples for the case of oxides and pure metals.

## 2. Experimental Investigations on Mixed Systems of Liposome and Nanoparticle

### 2.1. Role of the Lipid Bilayer Phase Behaviour

An important aspect for the understanding of the interactions of lipid bilayers with nanoparticles is the phase behaviour of the lipids. In this context it is important to note that the properties of a given bilayer membrane in the gel phase (L_β_) are extremely different from those it exhibits in the fluid phase (L_α_).

First of all the structural arrangement of the phospholipid molecules is different. As described earlier, in the gel phase lipid-acyl chains are conformationally highly ordered while in the fluid phase the bilayer possesses both translational disorder and a high degree of lipid-acyl-chain disorder. This implies that as the bilayer is heated from the gel to the fluid phase, its thickness decreases substantially due to the conformational changes experienced by the acyl-chains [30]. This decrease was analysed using both the Gibbs-Luzzati thickness (*D*_B_) model [44] (which partitions the overall bilayer thickness *D* into a lipid region *D*_B_ and a water region D_W_ thereby ignoring details of the interfacial region) as well as with the steric bilayer thickness (*D*_B_’) model (which takes into account the presence of water molecules mixed with the polar heads in the interfacial region) for the case of dipalmitoylphosphatidylcholine (DPPC) (Table 1). This work confirmed that this decrease is not model-dependent and typically in the range of 10% to 20% [45]. A concomitant increase in the bilayer area in the fluid state occurs, coming along with the corresponding decrease of the bilayer thickness, and that, of course, means one has then an enhanced hydration of the head group area. Similarly the state of the phospholipid chains has an important effect on the vesicle properties, in particular, when it comes to the deposition of supported lipid bilayers on nanoparticles where the surface ratio liposome/nanoparticle is of main interest. It can also be noted that the L_α_-phase shows a much larger swelling, which can be attributed to the undulation forces that are much more pronounced compared to the L_β_-phase (as the L_α_-phase possesses much lower bending moduli, see Table 2.), where the Helfrich undulation repulsion between two bilayers can be described by [46]:

(1) $$E_{und}(D)=\frac{3\pi^{2}}{128}\frac{{\left(kT\right)}^{2}}{\kappa_{b}D^{2}}$$

which gives the free energy of the steric interaction per unit area of membrane (*κ_b_*: mean bending modulus of one bilayer, *D*: distance between two adjacent membranes).

More importantly the fluidity of the membrane, as characterized by the bending elasticity, is dramatically altered by the phase transition. The bending elasticity (κ), described by the Helfrich theory [47] as the energy required to bend a bilayer away from its spontaneous curvature, determines the balance between adhesive and elastic forces responsible for the bending (and potential wrapping) of the bilayer around a particle [42,48]. Values of the bending elasticity for a given bilayer have been found to decrease drastically when the bilayer is heated through the phase transition to its fluid phase [49–51] (Table 2). This observation is related to the inability of the gel membrane to bend around particles or to fuse on flat surfaces, as a result of its high rigidity [52].

Finally, in the case of zwitterionic phospholipids, the electrostatic properties of the bilayer are also altered by the conformational changes occurring during the main phase transition. The dielectric permittivity of lipid bilayer, as well as its conductivity in aqueous solution, have been found to increase while passing from the gel to the fluid phase [53,54]. These changes, resulting from the decrease of the dipolar correlation taking place between the head group dipoles in the hydrophilic part of the bilayer, have a non-negligible influence on the surface interactions taking place between lipid bilayers and nanoparticles.

### 2.2. Nature of the Interaction in Mixed System Liposome/Nanoparticle

The interaction between lipid bilayers and solid surfaces is rather complex and includes van der Waals, double layer, hydration, hydrophobic, thermal undulation and protrusion forces [52]. The precise nature of the interaction, as well as the structure it creates, is in all cases related to the properties of the lipid and the solid surface used as well as the nature of the dispersing medium.

#### 2.2.1. Van der Waals Interaction

Describing the strength of van der Waals forces in mixed liposome/nanoparticle systems in a quantitative manner is challenging due to the geometry and nature of the system components. In the ideal case of a sphere (medium 1: nanoparticle) interacting with a shell (medium 3: liposome) in an aqueous medium (medium 2), the van der Waals energy is given by the equation [55,56]:

(2) $$E_{vdw}=-A_{123}\frac{R_{1}R_{2}}{6(R_{1}+R_{2})}\left(\frac{1}{d}-\frac{1}{\left(d+h\right)}\right)-\frac{A}{6}\text{ln}\left(\frac{d}{d+h}\right)$$

where *R*_1_ is the radius of the sphere (particle), *R*_2_ the outer radius of the shell, *h* the thickness of the shell, *d* the distance between the surfaces of the two objects and *A*_123_ the Hamaker constant of the system. An approximate value of *A*_123_ may be given in terms of the Hamaker constants of the individual media as follows [55,57]:

(3) $$A_{123}\approx (\sqrt{A_{11}}-\sqrt{A_{22}})(\sqrt{A_{33}}-\sqrt{A_{22}})$$

where *A_xx_* is the Hamaker constant between two semi-infinite planes of medium *x* in vacuum.

For “symmetrical” systems, the Hamaker constant is always positive, leading to attractive forces, but between dissimilar surfaces, as in the case of mixed systems liposome/nanoparticle, *A*_123_ can be either positive or negative leading respectively to attractive or repulsive forces. For phospholipid bilayer interacting with oxide particles (SiO_2_, TiO_2_) the Hamaker constant is typically (3 − 4) × 10^−21^ J ≈ 0.75 to 1 *kT* (for *T* = 25 °C) [52,58,59].

#### 2.2.2. Electrostatic Interaction

In general, the electrostatic double-layer interaction energy between two identical planar surfaces decreases exponentially with the distance. However, for two surfaces of different charge densities or potentials, which is the case in mixed system liposome/nanoparticle, this interaction energy can exhibit a minimum or a maximum at some finite distance [55]. The study on these asymmetric systems has led to different approximate equations for the interaction energy of two surfaces of unequal but constant potentials [60–62]. The “Hogg–Healy–Fuerstenau” equation (HHF) [55,60] for two different planar surfaces of low constant potentials *ψ*_1_ and *ψ*_2_ in 1:1 electrolyte is given by:

(4) $$W_{DL}(D)=\frac{{ɛ}_{0}ɛ\kappa [2\psi_{1}\psi_{2}-(\psi_{1}^{2}+\psi_{2}^{2})e^{-\kappa D}]}{\left(e^{+\kappa D}-e^{-\kappa D}\right)}$$

where *D* is the distance between the surfaces and κ their Debye length. This equation has recently been successfully implemented to characterize the double layer interaction between lipid bilayer and silica substrates [52].

Nevertheless, when working with nanoparticles and vesicles one often has to take into account the curvature of the interacting surfaces. To this aim, applying the Derjaguin approximation on equation 4 leads to the following formula for the approximate interaction energy between dissimilar double layers on two spherical particles with radii *R*_1_ and *R*_2_ [60]:

(5) $$W_{DL}(D)=\frac{ɛR_{1}R_{2}(\psi_{1}^{2}+\psi_{2}^{2})}{4(R_{1}+R_{2})}\left[\frac{2\psi_{1}\psi_{2}}{\left(\psi_{1}^{2}+\psi_{2}^{2}\right)}\text{ln}\left(\frac{1+e^{-\kappa D}}{1-e^{-\kappa D}}\right)+\text{ln}(1-e^{-2\kappa D})\right]$$

It is important to note that the HHF formula is based on the Debye-Hückel linear approximation. This approximation is thus applicable for sufficiently low potentials (commonly *ψ* < 25 mV). More recent work using the Poisson-Boltzmann expression for the potential has led to more accurate analytical approximations [61].

Alternatively, The Gouy-Chapman Theory has also been used to describe the electrostatic interaction energy between a flat substrate with surface potential *ψ*_sub_ and a flat bilayer of surface potential *ψ*_bil_ in monovalent salt solution [63,64] leading to the following formula:

(6) $$W_{DL}(D)=64kT\rho_{\infty }\;\text{tanh}\;\left(\frac{e\psi_{sub}}{4kT}\right)\;\text{tanh}\;\left(\frac{e\psi_{bil}}{4kT}\right)\frac{e^{-\kappa D}}{\kappa }$$

where *T* is the temperature, *k* the Boltzmann constant and *ρ_∞_* the ions concentration. This equation also assumes a constant surface potential at any distance *D*.

#### 2.2.3. Hydration Forces

In systems containing phospholipid bilayers, van der Waals and double layer interactions, as jointly expressed by the DLVO theory, often fail to precisely describe the phenomena taking place at short distances due to the presence of repulsive hydration forces [65,66], as has been observed experimentally by means of the surface force apparatus. These repulsive forces are believed to arise from the presence of a layer of water molecules, strongly bound to the hydrophilic surfaces and preventing them from approaching any closer than the thickness of two water molecules [55,65,67].

The full nature of this interaction is still an active field of research. However, in the case of hydrophilic surfaces in aqueous solution, the hydration energy is attributed to stable structured water layers hydrogen bonded to the solid surfaces, while the hydration repulsion between two lipid membranes is believed to be dominated by entropic factors (microscopic thermal fluctuations).

Tero *et al*. [64] were the first to propose an expression for the calculation of the hydration energy between lipid bilayer and hydrophilic surfaces, by assuming that the hydration between lipid membrane and solid substrate (*W*_hyd_) is the average of each hydration energy, giving:

(7) $$W_{hyd}=\frac{W_{solid}+W_{lipid}}{2}$$

where *W*_solid_ is the hydration force between hydrophilic substrates, depending on the nature of the substrate and its medium. Empirically, *W*_solid_ has been found to decay exponentially with the distance (*D*) between the surfaces [55,63,64]:

(8) $$W_{solid}=W_{0}e^{-D/\lambda_{0}}$$

where *λ*_0_ is on the order of 1 nm and *W_0_* depends on the hydration of the surfaces but is usually in the range 3–30 mJ·m^−2^ ≈ 0.7 to 7 *kT*·nm^−2^ (for *T* = 25 °C).

On the other hand, *W*_lipid_ is believed to arise from thermal fluctuations [68] and thus depends on the three different types of thermal motion that a membrane can experience: protrusion, peristalsis and undulation. The energetic contribution of these different motions can be separately expressed as follows [55,64]:

(9) $$W_{protrusion}=2.7\Gamma {kTe}^{-(\alpha_{p}D/kt)}$$

(10) $$W_{peristalsis}=\frac{{\left(kT\right)}^{2}}{20k_{a}D^{4}}$$

(11) $$W_{undulation}=\frac{3\pi^{2}{\left(kT\right)}^{2}}{64k_{b}D^{3}}$$

where Γ is the surface density of protruding head groups, *α_p_* the (hydrophobic) protrusion energy per unit length_,_ *k_a_* the area expansion modulus and *k_b_* the bending modulus. Typical values for these parameters are: *α_p_* = 2.5 × 10^−11^ J·m^−1^, *k_a_* = 0.15 J·m^−2^ and *k_b_* = 10^−19^ J [55,64] while Γ depends on the molecular occupying area of the chosen lipid.

It should be noted that undulation forces may be neglected in the case of membranes carrying unscreened surface charges or being under tension. Nevertheless, when considering every contribution, the hydration energy between a bilayer and a flat hydrophilic substrate is given by the expression:

(12) $$W_{hyd}=\frac{1}{2}\left\{W_{0}e^{-D/\lambda_{0}}+\frac{3\pi^{2}{\left(kT\right)}^{2}}{64\;k_{b}\;D^{3}}+\frac{{\left(kT\right)}^{2}}{20\;k_{a}\;D^{4}}+2.7\Gamma {kTe}^{-(\alpha_{0}D/kT)}\right\}$$

Additionally, it should be mentioned that the geometry of a curved surface tends to smother short range interaction (hydration) and emphasizes longer range van der Waals and electrostatic double layer forces [65,69,70]. This is of course of crucial importance when studying the balance of forces in mixed systems liposomes/nanoparticles where both species often exhibit a curved surface.

#### 2.2.4. Hydrophobic Interaction

Hydrophobic interactions may arise when phospholipid bilayers, subjected to a stretching force or stress, expand laterally and expose areas of their hydrophobic interior to the aqueous solvent.

Hydrophobic attraction plays an important role in the mechanisms of vesicle fusion or particle embedding, when hydrophobic particles are internalized into the hydrophobic interior of the membrane. In the case of vesicle-vesicle interaction, the increase in stress experienced by the membrane would be directly proportional to the increased adhesion force between two vesicles [55]. In the case of particle embedding, hydrophobic attraction can be expressed as the balance between the free energy change to move a hydrophobic sphere from pure water into a hydrophobic membrane (*ΔG*_emb_) and the energy penalty to deform the bilayer (*ΔG*_def_) [71]. Where *ΔG*_emb_ is given by [71,72]:

(13) $$\Delta G_{emb}=\pi D^{2}\gamma$$

where *D* is the nanoparticle diameter and γ the liquid-vapor surface tension of water.

Ultimately, the interplay between Van der Waals, double layer, hydration and hydrophobic interactions can be tuned by playing on numerous parameters, among which are pH, ionic strength and temperature, in order to obtain various structures such as supported lipid bilayers, internalized particles and decorated vesicles.

### 2.3. Structures Encountered in Liposome/Nanoparticle Systems

In the following we present selected different structures that can be obtained in membrane/nanoparticle systems and describe the balance of force contributions responsible for their formation and stabilization.

#### 2.3.1. Supported Lipid Bilayer (SLB) Formation & Particle Internalization

Supported lipid bilayers (SLB) are continuous fluid lipid membranes (*T > T*_m_) adsorbed on a solid substrate, and separated from this substrate by a thin water layer (1–3 nm) [73]. It is important to note the necessity of having a bilayer in the fluid phase (to have a membrane of sufficiently low elasticity [49–51]) and a hydrophilic substrate (to bind the supporting water layer) to achieve the deposition of SLB.

SLB formation has been studied first for the case of planar substrate (glass, silica, mica) as the basis to produce innovative catalytic surfaces or immobilized protein arrays [52,58,73–82], as well as to study bilayer-bilayer interactions, as they are important in biology, under well-defined conditions. Nowadays, this field of research has been extended to the case of nanoparticles, with the intention of designing nanovectors by rendering nanoparticles biocompatible through the deposition of a lipid bilayer onto their surface [83–85]. Such studies on well-defined membranes are important in order to control the interactions and thereby optimize the design of the delivery systems.

The formation of SLB, as has been investigated on both planar surfaces and nanoparticles, can be achieved by depositing vesicles from the solution [52,75,79,83–87] where the liposomes adhere, fuse and spread on the solid surface. In the case of a planar substrate, this process has even largely replaced the Langmuir-Blodget deposition due to the wider availability of more convenient methods for unilamellar liposomes preparation [76]. Despite the considerable work on vesicle adsorption, there is still no complete picture of the forces responsible for the adhesion, fusion and rupture of the liposomes on solid surfaces. However, the SLB formation is believed to be governed by three types of interactions [79,88]:

- vesicle-surface interaction;
- vesicle-vesicle interaction, as the lateral interaction between neighbouring vesicles adsorbed on a surface plays an important role in the process of vesicle fusion leading to SLB formation;
- cohesive strength of the vesicle (vesicle stability).

Note that the latter has been found to be of less importance in this process [88].

Experimentally, one can play with several parameters (ionic strength, addition of specific ions, pH, surface properties of the substrate, nature of the phospholipid(s) used) in order to monitor the strength of these three different interactions.

**Figure 1:**
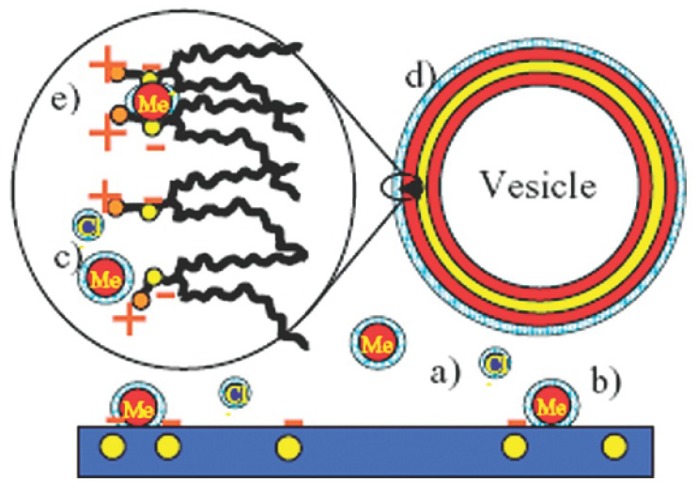
Schematic representation of system containing POPC (1-Palmitoyl-2-oleoylphosphatidylcholine) liposomes and a negatively charged silica substrate in a buffer solution. Cations and anions interact (**a**) with the water, forming a water shell more or less organized depending on the ion polarizability; (**b**) with the substrate surface, varying the surface charge density, and with the lipids, changing (**c**) the headgroup tilt; (**d**) the hydration properties, the intramembrane dipole potential (arrow), and (**e**) the nanomechanics via the packing and mutual interaction of lipids. At the studied pH (pH = 8), the surface of the SiO_2_ support is negatively charged. For this pH, the POPC lipid headgroups are zwitterionic, with the positive charge being slightly closer to the surface. Therefore, the lipid–surface interaction, in the absence of ions, could be regarded as that between a dipole (the lipid head), with its positive end closest to the surface, and a monopole (on the surface) carrying negative charge. However, the interaction is influenced by the presence of hydrated ions in the solution and especially for divalent ions the headgroup may be affected in the direction of being more like a monopole. Figure reprinted and adapted with permission from [88].

**Figure 2:**
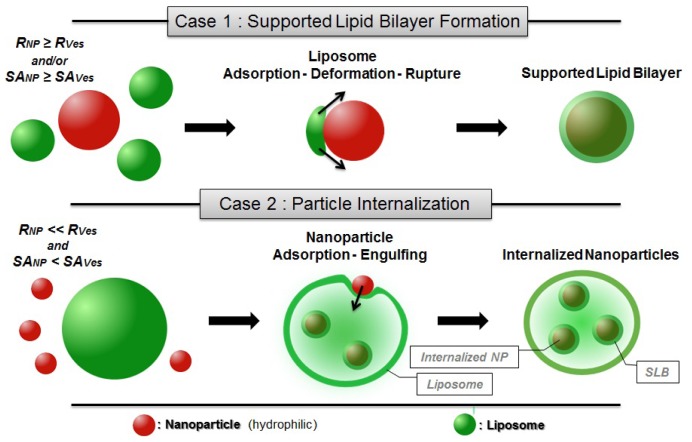
Schematic drawings of the possible mechanisms occurring in the mixed systems liposome/nanoparticle in the case of hydrophilic particles and fluid state liposomes, assuming sufficiently high attractive interactions between membranes and nanoparticles.

Changing the ionic strength and/or the pH is a way to alter the electrostatic properties of the substrate as well as the phospholipid net charge leading to different adsorption behaviors depending on the nature of the substrate and on the type of lipid used [52,75,84,85]. More precisely, the use of specific ions (in particular divalent cations) has been found to have a surprisingly strong effect on SLB formation, when bridging negatively charged or zwitterionic lipids and negatively charged substrates (silica-Figure 1) [79,86–89]. This is not surprising given the known affinity of Ca^2+^ and phosphate. The case of zwitterionic phospholipids is particularly interesting, as the cations convert the headgroup from a dipole toward a positive monopole thus strengthening the interaction with negatively charged substrate (Figure 2) [85,88].

However, even if the deposition mechanism is believed to be mainly driven by electrostatic attractions between vesicle membranes and the substrate surfaces, other forces are also non negligible. As an example, SLB formation or vesicle adsorption have been reported in systems where the electrostatic interactions were clearly repulsive [52] or minimized by experimental conditions (pH [75], substrate surface properties [90]) showing the importance of van der Waals attractive interactions as well as of hydration and steric forces in the deposition mechanism.

Previous studies on the formation of SLB on hard nanoparticles can be divided in two types that are depicted in Figure 2.

1. In the first case, the adsorption of liposomes onto nanoparticles leads to the formation of continuous SLB on the particle surface (Figure 2). This is the case when either the radius of the particle (*R_NP_*) is larger than the vesicle radius (*R_Ves_*) or when the surface area of all particles is equal or larger than the total surface of the potentially formed SLB (*SA_NP_* ≥ *SA_Ves_*—taking into account that when *SA_NP_* ≫ *SA_Ves_* nanoparticles are believed to be partially covered by lipid bilayer patches [91]).
2. In the second case, the formation of SLB on a particle is an intermediate step of the internalization mechanism in which particles covered with SLB become encapsulated within liposomes [42]. This may occur when *R_Ves_* is considerably larger than *R_NP_* and *SA_NP_* inferior to *SA_Ves_* (Figure 2). The internalization of the nanoparticles is firstly driven by the attractive interaction between membrane and nanoparticle but in addition, a thermodynamic driving force is the release of water from the vesicle interior and the concomitant entropy gain that occurs during the process.

(1) In the first case mentioned above, the mechanism of SLB formation proceeds via subsequent adsorption, deformation and rupture of liposomes on the nanoparticle surface [83] much like in the case of flat substrates [52,79,80,89]. After adsorption of the liposomes, the contact area between the lipid surface and the substrate increases, due to adhesion forces until the deformation of the vesicle reaches a limit above which rupture occurs, resulting in the appearance of patches on the particle surface. It is to be noted that this process has been found to occur either independently or dependently of the presence of preformed lipid patches in the vicinity of the adsorbed vesicle. As a matter of fact, the edges of bilayer patches are likely to activate the decomposition of adsorbed vesicles [79]. Hence, the formation of a SLB covering an entire particle often involves the coalescence of neighbouring liposome patches via active edge effects [89]. In any case the binding of a nanoparticle to a bilayer will lead to a loss of configurational entropy of the membrane as it suppresses undulations of the bilayers.

However, the process of SLB formation depends highly on the ability of the membrane to curve around the particle and adopt its shape [92,93]. The curvature of the lipid bilayer is depicted in the literature as the result of the interplay between the (favourable) adhesion energy between the membrane and its substrate and the (unfavourable) elastic energy (or bending energy) needed to bend the bilayer around the particle [48,80,89,92–94]. Corresponding theoretical studies have led to structural phase diagrams demarking the regions of fully enveloped, partially wrapped or freely dispersed colloids [95–97]. The investigations on the transition steps between the wrapped and the free state of particles allow the definition of a critical radius for the particle (*R_C_*), meaning that the formation of SLB is energetically favourable on particles having a radius above this critical value. In the case of a tensionless membrane, the value of *R_C_* may be extrapolated from the expression of the energetic balance when the adhesion energy is compensated by the bending energy [48,98], giving:

(14) $$R_{C}\equiv \sqrt{2\kappa /|W|}$$

where *R_C_* depends on the strength of the adhesion energy per unit area (*W*) as well as on the value of the bilayer mean bending modulus (κ), thus depending on the nature of both particle and lipid bilayer. Consequently, one can modify this critical value by changing experimental conditions such as the nature of the lipids, the properties of the substrate surface [90] as well as the properties of the medium such as pH [84] and ionic strength [85]. For instance the bending modulus (κ) is proportional to the third power of the bilayer thickness [99–101]. Accordingly, the values of *R_C_* determined experimentally have been found to vary with the nature of the system. In the case of silica particles interacting with zwitterionic lipid bilayers, the critical radius has been found to be around 10 nm [42,92,93] corresponding to an adhesive strength *W* of a few mJ/m^2^—a value comparable to usual interfacial energies [55].

**Figure 3:**
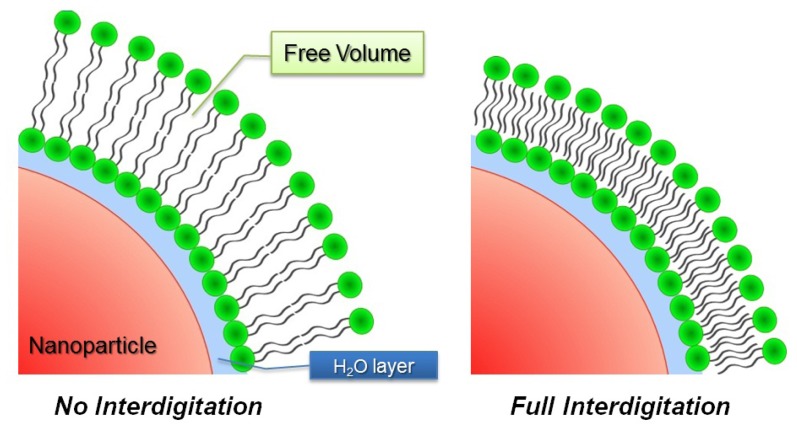
Schematic drawings of the interdigitation of the outer and inner leaflet of a phospholipid bilayer supported on a small nanoparticle.

Interestingly, supported lipid bilayers adsorbed on small particles (*R_C_* < 100 nm) exhibit morphology changes due to the high curvature they experience. Shifts of the *T*_m_ value [102] as well as a broadening and/or splitting of the transition peak obtained by differential scanning calorimetry (DSC) [102,103] suggest a curvature-dependent decoupling between the inner and outer leaflet of the bilayer as has also been observed in the case of small SUVs [104,105]. In extreme cases (*R_P_* ≤ 10 nm) the high curvature induces the creation of a large free volume between the acyl-chains of the supported bilayer [102,106] (Figure 3, left). In this case, interdigitation of the acyl-chains occurs in order to optimize hydrophobic interaction while avoiding the exposure of hydrophobic chains to an aqueous environment (Figure 3, right) [107].

**Figure 4:**
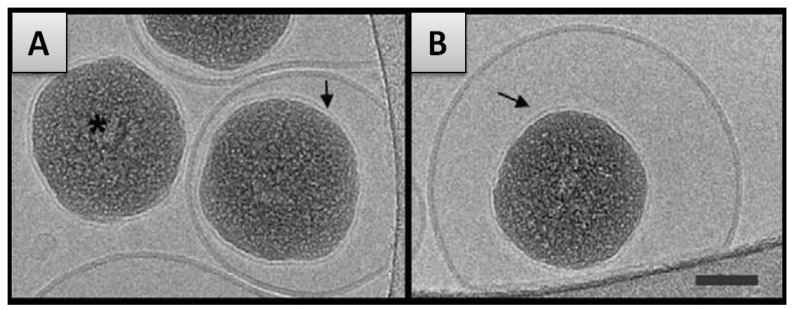
CryoTEM images of internalized silica hard nanoparticles (NPs) within DOPC (dioleylphosphatidylcholine) liposomes—visualization of the SLB on internalized particles (black arrow) and of “free” NP covered only with a SLB (asterisk). Scale bar 50 nm. Figure reprinted from [42].

(2) In the second case mentioned previously, the SLB formation on smaller particles is accompanied by the internalization of the nanoparticle into the vesicle interior, as has for instance been observed for the case of silica NPs interacting with DOPC vesicles (Figure 4). As described by Le Bihan *et al*. [42], the proposed invagination mechanism predicts the adsorption of the particle on the liposome outer surface, followed by the spreading of the membrane around the particle finally leading to the engulfing and the full internalization of the nanoparticle. This process of nanoparticle transmigration is similar to that of cellular uptake (or endocytosis) [108,109]. Simulations on similar systems [110] have been found to consistently reproduce the invagination mechanism described above.

The invagination mechanism is governed by the same energy balance as in the previously discussed case of SLB formation [48,49,96,111]. Accordingly, the same expression (Equation 14) arises for the calculation of the critical particle radius value below which the particle remains adsorbed on the liposome outer surface. In this case, the total adhesion energy does not overcome the energy cost associated with the bending of the membrane, thus preventing the invagination mechanism to proceed further. Nevertheless, the experimental findings [42] seem to contradict some of the requirements expressed by theoretical studies such as the necessity of working with large liposomes (*R_Ves_* > 300 nm) [95] as well as the required presence of rafts which are phase separated from the rest of the membrane in order to promote the fission process allowing the detachment of the internalized particle from the membrane [111]. However, related to the latter, it may not be necessary to tune the membrane to obtain phase separated domains since the adsorption of particles has been found, in some cases, to induce local phase separation on its own [112,113].

#### 2.3.2. Decoration and Aggregate Formation

**Figure 5:**
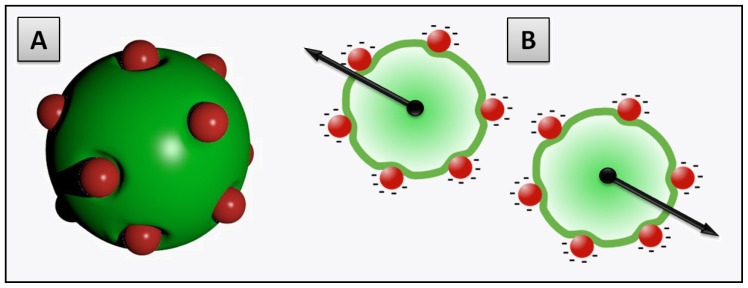
Schematic drawing of a decorated vesicle structure featuring the local membrane bending caused by the particle adsorption (to scale [40]) (**A**) and the electrostatic repulsion (black arrows) between the adsorbed charged nanoparticles responsible for the colloidal stabilization of the liposome dispersion (**B**).

Vesicle decoration results from the adsorption of hydrophilic NPs onto the surface of liposome membranes in systems where the nanoparticle radius is below the critical value *R_C_* (Equation 14) [40,43,114] (keeping in mind that this value can be controlled by tuning other parameters such as pH or ionic strength of the solution) [43]. In this case, the balance between adhesion and bending energy is therefore shifted to a range where it is unfavourable to the invagination process. Thus, the membrane is “incapable” of internalizing the particles, leaving them on its outer surface (Figure 5A). Due to the attractive interaction between membrane and the nanoparticles there will be indentations by the NPs into the membrane surface which depend on the balance between attractive interaction and the bending energy required for this partial wrapping. This does not only lead to a deformation of the membrane but affects also its local stiffness and will moreover cause an additional interaction force between the different NPs attached. Furthermore it has to be considered, that in some cases, where the particle radius is close to the critical value, the kinetic of the invagination process is slowed down, allowing the observation of decorated vesicle structures which, in these cases, are only intermediate structures [115].

For a number of cases of charged nanoparticles, the decoration of liposomes by particles (zwitterionic lipids/charged nanoparticles) has been found to increase the absolute Zeta potential value of the dispersion by introducing charges on the liposome surface [40,43]. The appearance of these charges on the vesicle surface gives rise to repulsive electrostatic forces between the decorated vesicle structures and thus stabilizes the vesicle dispersion [40,41,43]. This repulsion can be quantified by means of measurements of the ζ-potential and such experiments have shown a decrease of the ζ-potential and concomitant stability increase which becomes larger with increasing nanoparticle sizes [43]. This result is of great interest given the metastable nature of many liposome dispersions [35] which often leads to rather fast phase separation and which represents a major disadvantage for their potential applications. Accordingly, the addition of charged silica NPs is a way to regulate the stability of liposomes.

However, in these systems, the colloidal stabilization is only achieved upon the adsorption of a minimum amount of nanoparticles, which corresponds to a sufficient surface coverage of the liposomes to ensure the repulsion between the decorated vesicle structures (Figure 5B) [40,41].

**Figure 6:**
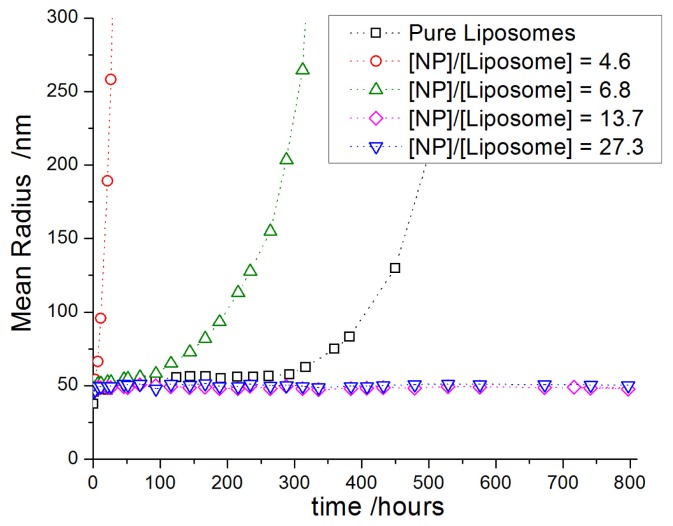
Evolution of the hydrodynamic radius of dipalmitoylphosphatidylcholine (DPPC) vesicles (*R*_h_ ≈ 40 nm) decorated with different amount of silica NP (*R*_h_ ≈ 8 nm) in water for various ratios of nanoparticle per vesicle (experiment done at 25 °C).

This has similarly been observed in recent work by us as documented by the change of particle size as a function of time (Figure 6). This study on the stability of unilamellar DPPC vesicles (R ~ 50 nm) below their phase transition temperature by means of dynamic light scattering showed that the addition of small amounts of small silica NPs (*R*_h_ ≈ 8 nm) leads to a rapid destabilization of the vesicle dispersion [40]. Only the adsorption of a sufficiently high amount of silica particles onto the liposome surface, here about 14 NPs per vesicle, is able to prevent the fusion of neighbouring liposomes and ensure their colloidal stability for several months. This is an enormous enhancement compared to the situation of the unmodified vesicles and such long-time stability is very important for the storage of liposome dispersions and also for having controlled release properties.

**Figure 7:**
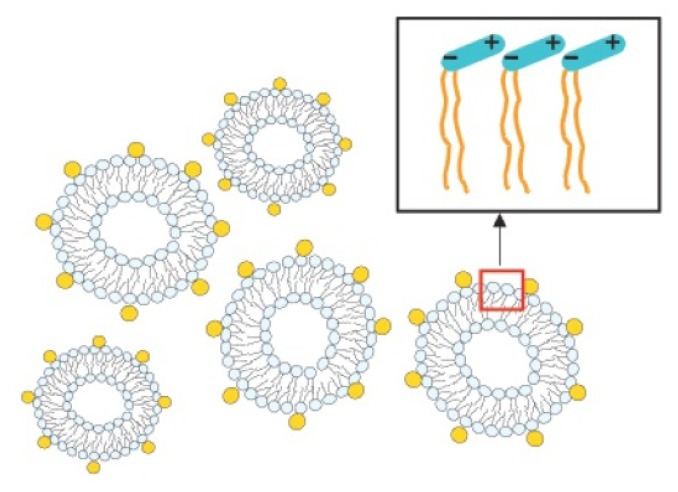
Schematic illustration of a DLPC (dilauroylphosphatidylcholine) liposome suspension stabilized by nanoparticles (not to scale). The inset shows a schematic diagram of the P^−^–N^+^ dipolar headgroup of a zwitterionic lipid. Therefore, an anionic nanoparticle, with electric charge opposite to that of the outermost portion of the lipid headgroup adsorbs more strongly than a cationic nanoparticle. Figure reprinted and adapted with permission from [114].

On the contrary, if the NP concentration is too low, nanoparticles would bridge between adjacent liposomes, thereby introducing interactions and thus accelerating their fusion [40,41], as has been observed in nanoparticle-microsphere systems [116]. In order to limit the magnitude of this effect, one may change the sign and magnitude of the particle charge. As has been found for PC liposomes, the use of cationic particles allows colloidal stabilization at lower surface coverage as compared to anionic ones [114]. In fact, cationic particles adsorb weakly on the membrane due to the geometry of the P^−^–N^+^ dipole of the lecithin head group (Figure 7), and consequently, they are less apt to bridge between neighbouring liposomes. These results confirm the predictions made by computer simulations on analogous systems [117,118].

Specific studies on these nanoparticle-stabilized liposomes have led to interesting findings on their properties. First and in opposition to other stabilization methods (PEG coating [119–121]), the adsorption of nanoparticles neither prevents nor reduces the binding between liposome immobilized ligand (biotin) and free receptor proteins (streptavidin) in the bulk: at low surface coverage (but still sufficient to ensure colloidal stabilization), the outer surface of decorated liposomes has been found to be well accessible and biofunctional despite the presence of NPs [122]. Furthermore, the particle decoration does not seem to prevent its permeability; on the contrary, the release of molecules (proteins) loaded in the aqueous interior of the liposome may be more accurately controlled by appropriately engineering the hydrophilic nanoparticle layer on the liposome outer surface [123]. This is important for potential applications, since it allows for the stabilization of liposomes while retaining their other (biological) functionalities. Additionally, decorated vesicles are easily immobilized on a planar substrate by tuning the substrate charges to create an attractive electrostatic interaction between the substrate and the adsorbed nanoparticles [124]. Such immobilized liposomes have an amazing potential as nanocontainers for the study of biomolecules: they allow the prolonged observation of molecules loaded in their inside with lesser surface perturbation than in the bulk.

#### 2.3.3. Internalization within the Membrane

The incorporation of nanoparticles within the bilayer membrane occurs when using hydrophobic nanoparticles which have a higher affinity to the acyl-chains confined in the hydrophobic interior of the phospholipid bilayer. The most common preparation pathway to form such vesicle-nanoparticle hybrids is to dry a mixture of hydrophobic particles and phospholipid MLVs in chloroform, thus obtaining a NP-lipid film which is hydrated and further extruded or sonicated to obtain the final Vesicle-NP hybrid dispersion—provided the nanoparticles are sufficiently small [125].

There are numerous studies on such loaded vesicles using different hydrophobic particles including Au [71,126–129], Ag [130,131], Si [125], Fe_3_O_4_ [132,133], and quantum dots (CdSe, ZnS) [128,130,134,135]. In most cases, the incorporation of nanoparticles does not appear to have a significant influence on the vesicle size or stability [125,136] even though there has been mention of a decrease of the liposome size upon particle embedding for which no explanation has yet been provided [128,133]. However, the internalized NPs will induce local disruption of the bilayer structure, reduce lipid ordering and thereby grant a higher fluidity to the membrane [126,131,136] at the same time changing the thermodynamics of the lipid bilayer phase transition (merging of the pretransition–transition from the ordered gel (L_β_) to the rippled gel phase (P_β_)–with the main phase transition, and a broadening of the melting region and shifts in *T*_m_) [129,130,136,137].

Theoretical studies have defined the mechanism of particle embedding as the result of the dominance of the favourable free energy change caused by moving a hydrophobic sphere from pure water into the hydrophobic membrane over the energy penalty needed to deform the bilayer [138,139]. In that context, it might be noted that the gain in interfacial energy (Δλ·A) is about 1000 k*T* for a nanoparticle of 4 nm radius (for a change of interfacial tension of 20 mN/m). Nevertheless, the incorporation within the membrane is only energetically favourable if the particle size is inferior to a critical size depending on the type of lipid used and being close to the value of the membrane thickness [71,125,138,139]. Larger particles cannot become accommodated within the lipid bilayer due to the high elastic energy penalty, which then would suppress the membrane fluctuations, and the geometric difficulties of having a membrane surrounding such a large nanoparticle. Yet the interaction of liposomes or phospholipid molecules with large and highly hydrophobic particles (as well as with flat hydrophobic substrates) has been reported to lead to the formation of a phospholipid monolayer on the hydrophobic surfaces [140–143]. The resulting structures may be used as biocompatible particles for drug delivery [141,144,145], as column materials for high-pressure liquid chromatography [146] or else as a way of investigating membrane-like assembly in an immobilized state [140,142,147].

**Figure 8:**
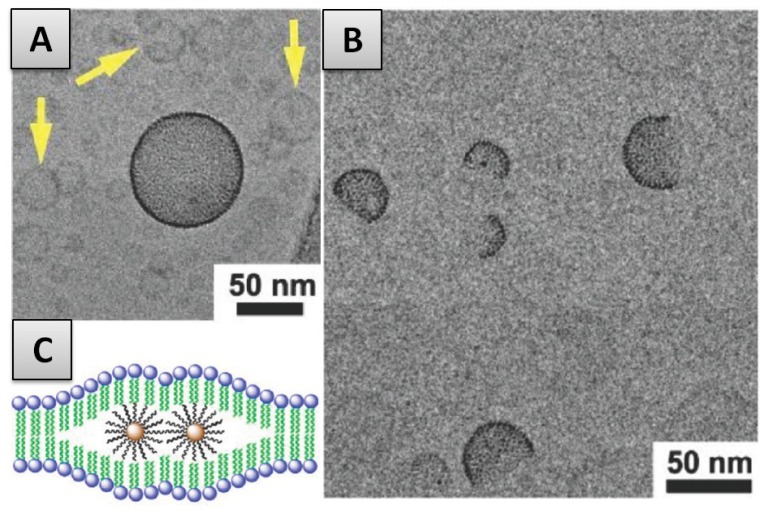
Membrane Internalization. (**A**)-CryoTEM image of a Vesicle-NP hybrid surrounded by normal small unilamellar vesicles (SUVs); (**B**)-CryoTEM image of janus vesicles; (**C**)-Schematic illustration of membrane-internalized hydrophobic NP clustering inside the membrane. Figure reprinted and adapted with permission from [71].

However, in the case of the internalization of small NPs in the hydrophobic interior of the phospholipid bilayer, the phospholipid membrane must “unzip”, *i.e.*, dissociate the acyl-chains of both layers (Figure 8C). The unzipping creates void spaces in the vicinity of the incorporated NP, an energetically unfavourable situation. In order to minimize this void space as well as the free energy of deformation, incorporated NPs have been found to cluster within the bilayer (Figure 8C) [71,129] resulting in the appearance of either a few fully loaded liposomes (vesicle in the center with dark rim) within a “normal” SUV dispersion (Figure 8A) of vesicles without NPs contained in the membrane. In other specific cases, Janus-type vesicle structures are formed where internalized NPs are concentrated on one side of the vesicle (Figure 8B) [71]. Very recent work on this phenomenon has underlined the importance of the bilayer phase behaviour in this process of particle aggregation. Nanoparticle clustering is observed upon lipid melting, and believed to be driven by greater hydrophobic mismatch between embedded particles in the fluid phase coupled with lipid mediated forces driven by lateral capillarity [129]. Nonetheless, although the argument justifying the clustering of particles is quite convincing, the preferential embedding of particles in a few vesicles within a liposome dispersion or in one side of a vesicle (case of Janus-vesicles) has not been reported in other similar works [130,133,135] and therefore is still a topic open for discussion.

Such nanoparticle-vesicle hybrids are promising tools in nanobiotechnology. For example, the use of quantum dot-vesicles allows the controlled vesicle fusion on cell surfaces while at the same time enabling the visualization of the resulting patches on the cell membrane by fluorescence [135]. Also, embedded paramagnetic nanoparticles, when heated using an electromagnetic field, have been found to provide a useful means of controlling the liposome membrane permeability by inducing bilayer phase transition [133,137,148]. One important challenge of this novel technology is to achieve the phase transition locally, without changing the temperature of the medium which, *in vivo*, may damage the biological structures in the vicinity of the NP loaded liposome (this is also an important challenge in the case of the light actuation of gold NP, see Section 3.2.). To this end, unlike the case of SPIO (super paramagnetic iron oxide) particles [137], the use of individually stabilized iron oxide particles, has been reported to allow local heating of the membrane without major influences on its environment [133]. This system then allows for directed and controlled delivery of active agents from vesicle systems. In addition, of course, this approach allows for a much lower and controlled energy input, which is a desired effect, especially when considering potential medical applications.

## 3. Applications

Combining the properties of phospholipid vesicles and nanoparticles as well as observing the influence of nanoparticles on biological membranes for which liposomes may serve as a model system leads to innovative approaches for novel medical applications. In addition, they are a relevant model system for investigations regarding nanotoxicity [149], since nanoparticles have received increased scrutiny and attention with respect to their potential effects on health issues. Furthermore nanoparticles can serve also as formulating agents to control the properties of membranes since they are frequently employed in vesicle formulations as applied in pharmacy or cosmetics.

### 3.1. Oxide Nanoparticles (Silica)

Before being used for medical purposes, NPs were subjected to numerous preventive studies about their cytotoxicity [150–153]. In this respect, silica particles appear to be a preferential choice since silica is considered as a biocompatible material due to its low cytotoxicity [150] and is furthermore required for the production of structural material of many living organisms [154]. However, more than its low toxicity, the versatility of silica in synthesis as well as in surface functionalization is the main argument for the use of silica particles in biotechnology [10]. As a matter of fact, silica particles can be easily synthesized in various ways [9,155,156] and the silanol groups on their surface can react with various compounds to form amine, carboxyl, thiol, or other groups, *i.e.*, there exists a large versatility of possible chemical surface modifications. However, silica surface modification can also be performed by passive adsorption of molecules such as proteins, [10,12] granting silica particles an amazing potential as very versatile drug carriers.

More specifically, mesoporous silica nanoparticles (MSN) have been found very attractive for drug delivery applications due to their high surface area and their tuneable pores [157–160]. In such particles, the loading of drugs can be achieved up to very high concentrations through simple electrostatic interactions. Nevertheless, once in the body fluids, drugs in accessible pores may be displaced by metabolites/ions leading to premature release. To avoid this phenomenon, gating devices have been designed using polymers [161] or nanoparticles [162,163] to regulate the release of guest molecules. In this precise matter, supported lipid bilayer (SLB) adsorbed on mesoporous silica particles has been found to be very efficient as a gating agent while at the same time ensuring the biocompatibility of the particles and facilitating its cellular uptake [164]. Furthermore the surface charges of such protocells are easily tuned by lipid exchange when introducing free liposomes of different composition in the bulk [164].

Similarly, in the case of plain silica particles, lipid bilayers supported on silica NPs have been found to be a useful transfection agent for gene delivery [165]. In this case, SLBs present many advantages over empty liposomes: SLBs exhibit a higher stability, since the lipid layers are unlikely to fuse due to the presence of a solid core, while allowing the monitoring of the SLB size by changing the size of the silica core.

Decorated vesicle structures based on silica particles have also been shown to have interesting features for medical application. In fact, adsorption of negatively charged silica nanoparticles on the vesicle membrane stabilizes the liposome dispersion by introducing electrostatic repulsion between the particle/vesicle complexes while retaining the fundamental properties of free liposomes [41,123]. Furthermore, when adsorbed on drug loaded liposomes travelling in a biological media, the nanoparticulate outer layer protects the liposome against enzymatic degradation and at the same time allows for the controlled release in gastric conditions [123]. Similar results have been obtained with liposil (silica/phospholipid composite) dispersions, where the liposome is used as template for the hydrolysis and condensation of TEOS leading to the synthesis of a protecting silica nanocapsule around the liposome [166–168]. Further studies have been carried out on the deposition of supported lipid bilayers on liposils leading to liposome-silica-liposome structures where the two bilayers are separated from each other by a sol-gel derived silica layer [169]. These hybrid structures present a significant interest since they allow the separated formulation of each of the two membranes to be optimized for their given purposes increasing the control over the drug release mechanism.

### 3.2. Metallic Nanoparticles (Gold and Silver)

**Figure 9:**
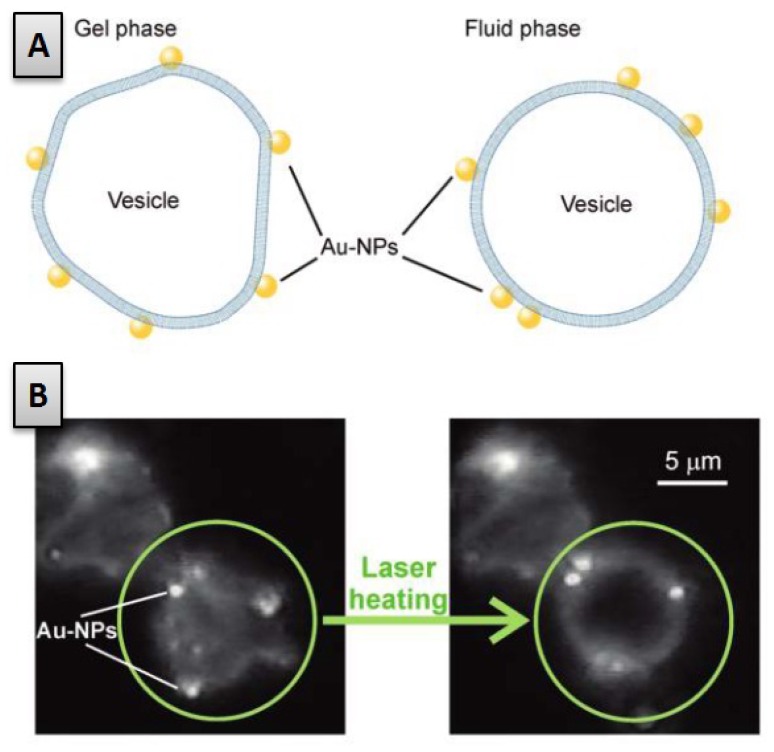
Optically induced phase transition of a AuNP-modified giant unilamellar vesicle. (**A**)-Schematic of the phenomenon (**B**)-Dark-field micrograph of two adjacent gel-phase vesicles modified with gold nanoparticles. The lower vesicle illuminated with the heating laser relaxes to a spherical shape. Figure reprinted with permission from [170].

Metallic nanoparticles, for instance made from gold and silver are rather frequently employed, where for Au NPs a high potential for catalytic properties exists and Ag NPs are employed in many applications due to their antimicrobial properties. Accordingly, the impact of such metal NPs on lipid bilayer presents many interesting aspects with respect to their nanotoxicity and also for further medical applications of such metal NPs. One interesting feature of metallic particles (especially Au nanoparticles) is their ability to efficiently convert electromagnetic radiation into heat. Hence, the irradiation of Au nanoparticles staying in the vicinity of a lipid membrane has been found to induce locally reversible phase transition in lipid bilayer systems by means of a laser heating. As shown in Figure 9 this phase transition can be observed optically as it may lead from facetted vesicles in the gel state to spherical vesicle in the fluid state [170]. By monitoring the size of the nanoparticles and the energy input by irradiation one can control the exact particle temperature while at the same time controlling the size of the melted footprint on the bilayer around the irradiated particle [171]. Such an optical control over the gel-fluid phase transition has also been reported to allow the guiding of a nanoparticle along the membrane plane [170]. In fact, although Au nanoparticles coated with CTAB are immobile when attached to a lipid bilayer in the gel phase, when laser heated, the particle induces the melting of the membrane in its vicinity, granting the lipids a much higher mobility thereby re-enabling its own motion. By tuning the properties of the irradiation one can achieve an effective guiding of the particle along the bilayer [170], thus enhancing the potential of single nanoparticles as thermally controlled nanotools.

These features of Au nanoparticles may have a great impact in medical applications as is the case for the remote-release of molecules from the interior of the liposomes [172]. In this instance, optical heating on liposome-NP assemblies, with adsorbed [172] or embedded particles [127], has been found to remotely release dye molecules from the interior of the liposomes by inducing local phase transition. An extensive use of the technique of optical heating has led to the development of a new approach in cancer therapy: the so-called selective nanophotothermolysis [173,174]. Here, the selective attachment of metallic nanoparticle on cancer cells is achieved by tuning the surface properties of the NPs (coating with antibodies) [175] in order to achieve the selective delivery properties. The irradiation is then tuned so that the temperature of the NP exceeds the threshold of bubble formation in the surrounding medium, thereby creating vapour bubbles which disrupt and destroy the cell membrane.

One of the main remaining challenges of these medical applications is the ability to perform the irradiation without damaging the environmental tissues and cells. This could be achieved by choosing a wavelength in the near-IR part of the spectrum [172,176], where light is only absorbed weakly by biological molecules and thus exhibits a deep tissue penetration. However, small single Au NPs (with diameter inferior to 200 nm) exhibit a surface plasmon resonance in the visible part of the spectrum (≈530 nm) [170,172] while not absorbing substantially at wavelengths closer to the IR-domain (illumination at 633 nm) [170] thereby preventing the use of optical heating in the near IR-domain. In this precise case the use of larger gold particles or gold NP aggregates as well as gold nanorods, which have been found to lead to higher near-IR absorption [172,177], is a way to efficiently achieve selective optical heating.

It is also of major interest to consider the case of silver particles due to their increasing mass production and domestic use [178].

Silver nanoparticles (Ag-NPs) may be used as biosensor material [179] but are mostly interesting for their antibacterial [7,180,181] and antiviral properties, as they have been found to inhibit the HIV-1 virus infectivity *in vitro* [182,183]. Silver nanoparticles are thus intensively used in a wide range of applications. For example in surface treatment, when deposited on polymer and metal surfaces [184] as well as in the textile industry [185] where wool textiles treated with nanosilver colloidal solution develop various functionalities such as mothproofing, antibacterial and antistatic properties. However, applications of Ag-NPs as an anti-proliferative agent could be limited by the fact that they are equally toxic to human cells and DNA [186–188]. In this context hybrid lipid/nanoparticle conjugates provide a biologically inspired route of designing therapeutic agents and a means of reducing and controlling nanoparticle toxicity.

In one pathway of producing such hybrid materials, liposomes are used as nanoreactors, where the nucleation and growth of Ag-NP takes place, as well as a stabilizing agent forming an SLB around the silver particle surface and preventing their aggregation [189]. This stabilization occurs without dramatically altering antibacterial properties of the nanoparticles. Nevertheless, an excessive increase of the lipid concentration may lead to the presence of multiple lipid bilayers on the particle surface which then results in a lower bacterial killing rate [189,190]. Similar work has been carried out using nanotubes consisting of Ag-NP embedded bilayer membranes where the spacing between bilayer membranes is used as scaffold for the growth of Ag-NPs. Similarly, in this case, the bilayer membrane contributes to stabilizing the NPs against aggregation and air oxidation processes while controlling their size [191].

Another route arises when using hydrophobic decane-thiol or stearylamine modified Ag-NPs. Due to their higher affinity to the hydrophobic interior of the lipid bilayer, these hydrophobic NPs are embedded into the membrane provided their size is equal or less than the bilayer thickness (See Section 2.3.3.). Similar to the case of embedded gold or SPIO NPs [126,137] the presence of Ag-NPs within the membrane can induce changes in lipid packing and may disrupt the lipid-lipid interactions leading to changes in the bilayer phase behavior and viscosity. Studies on such systems have revealed a suppression of the lipid pretransition, a broadening of the main phase transition region [136] as well as an increase in membrane fluidity above the *T*_m_ [131] when an important amount of Ag-NPs is internalized into the membrane. Thus, depending on their size and surface chemistry, embedded NPs may have an influence on the stability and function of hybrid vesicles providing innovative routes to designing drug delivery systems.

## 4. Conclusions

The investigations of the interaction of nanoparticles with vesicles (liposomes) as model systems for membranes cover a very variable field of recent research activities, as this is one of the key aspects for understanding nanotoxicity. This is important as nanoparticles have recently been employed increasingly in a variety of different applications due to their specific properties. Furthermore nanoparticles can also be utilized as transport vehicles for selectively delivering active agents into cells where their interaction with the membrane is a crucial aspect in controlling the delivery properties. Nanoparticles can bind strongly to the membrane surface but also become incorporated into bilayers or transported through the bilayers. The latter process is an essential step in the context of nanotoxicology as the incorporation of nanoparticles in cells is an elementary stage in this process, and may depend largely on pH, ionic strength and, of course, on the detailed structure of the nanoparticles, that can be changed by chemical or physical modifications. Alternatively the selective incorporation of nanoparticles into membranes is one way to introduce a sensor into the system or to obtain a means of controlling properties such as permeability of membranes via light induced heating. In summary, it can be stated that nanoparticle/liposome interactions are the key for understanding many developments in nanomedical research.

## Figures and Tables

**Table 1:** Parameters that characterize the structure of DPPC (dipalmitoylphosphatidylcholine) bilayers in the liquid phase (50 °C) and in the gel phase (20 °C). *V*_L_: lipid molecular volume. *D*: lamellar repeat spacing. *A*: average interfacial area per lipid. *D*_B_: Gibbs-Luzzati bilayer thickness. *D*_W_: Gibbs-Luzzati Water thickness. *D*_B_’: steric bilayer thickness. *D*_W_’: steric water thickness. Figure reprinted and adapted with permission from [45].

Temperature/°C	20	50
*V*_L_/Å^3^	1144	1232
*D*/Å	63.5	67
*A*/Å^2^	47.9	64
*D*_B_/Å	47.8	38.5
*D*_W_/Å	15.7	28.5
*D*_B_’/Å	42.4	46.5
*D_W_*’/Å	11.1	20.5

**Table 2:** Bending elasticity *κ_c_* of saturated bilayers (PC: phosphatidylcholine) in D_2_O at different temperatures, measured with the neutron spin-echo (NSE) technique. Figure reprinted and adapted with permission from [51].

Lipid	*T*_m_/°C	*T*/°C	(*T − T*_m_)/°C	κ*_c_*/*k_B_T*
14:0 PC	24	22	−2	100.0 ± 4.99
		24	0	20.9 ± 0.61
		28	+4	13.9 ± 0.24
		35	+11	15.3 ± 0.31
		45	+21	13.9 ± 0.44
		60	+36	8.2 ± 0.12
16:0 PC	41	30	−11	49.6 ± 2.78
		41	0	36.1 ± 1.49
		60	+19	9.5 ± 0.18
18:0 PC	54	40	−14	79.1 ± 3.23
		60	+6	13.6 ± 0.24
